# The Performance-based IISCA Can Inform Effective and Socially Meaningful Skill-based Treatment

**DOI:** 10.1007/s40617-024-01036-7

**Published:** 2025-01-08

**Authors:** Tess Fruchtman, Joshua Jessel, Bai Pan, Shauntae McLeod, Adithyan Rajaraman

**Affiliations:** 1 Department of Psychology, Queens College, City University of New York, Queens, NY USA; 2 Department of Teaching and Learning, College of Education and Human Development, Temple University, Philadelphia, PA USA; 3 Department of Applied Disability Studies, Brock University, St. Catharines, ON Canada; 4 Department of Pediatrics at Vanderbilt University Medical Center, Nashville, TN USA; 5 Vanderbilt Kennedy Center’s Treatment and Research Institute for Autism Spectrum Disorders, Nashville, TN USA

**Keywords:** Functional analysis, Challenging behavior, Synthesized contingencies, Trauma-informed care

## Abstract

Jessel et al. (*Behavior Analysis in Practice, 17*, 727–745, 2024) demonstrated that results from the performance-based, interview-informed synthesized contingency analysis (IISCA) had strong correspondence when compared to typical IISCA procedures and produced positive outcomes with resultant functional communication training procedures. On the basis of the assumption that functional analyses may include potentially adverse events insofar as they deliberately and repeatedly arrange conditions suspected to evoke dangerous behavior, Jessel and colleagues argued in favor of aligning functional analysis procedures with guidelines of trauma-informed care. We replicated and extended Jessel et al. (*Behavior Analysis in Practice, 17*, 727–745, 2024) by conducting a performance-based IISCA with three children with autism referred for behavioral services due to dangerous behavior and by evaluating a comprehensive skill-based treatment informed by the performance-based IISCA. The skill-based treatment resulted in the eventual elimination of dangerous behavior and the acquisition of multiple important skills, with caregivers implementing treatment sessions for two of the three participants. Assessment and intervention procedures and outcomes were socially validated by all participating families.

In addition to certain core behavioral characteristics (e.g., deficits in communication skills and social interactions, restrictive and repetitive patterns of behavior) associated with autism spectrum disorder (ASD), challenging behavior is common among children with autism (Blumberg et al., 2013). How challenging behavior is defined can vary; however, dangerous topographies can include disruptive behavior, aggression, and self-injurious behavior (SIB). If a child with autism presents with dangerous behavior, it can lead to difficulty succeeding in school (Estes et al., 2011), ostracism from the community (Turnbull & Ruef, 1997), and a higher likelihood of being placed in a residential facility (Matson et al., 2011). Challenging behavior is by no means specific to ASD; however, many behavior analytic procedures have been developed to address the challenging behavior of individuals with autism.

The practical functional assessment (PFA; Hanley et al., 2014) model for addressing challenging behavior focuses on identifying the problematic context in which the behavior occurs. The PFA begins with an open-ended interview with caregivers, therapists, teachers, or any adult familiar with the challenging behavior exhibited by the individual. Throughout the interview, the clinician asks questions about the topographies of challenging behavior, as well as the variables likely to turn the challenging behavior “on and off.” Using the information gained from the interviews, the clinician designs and conducts an individualized functional analysis using synthesized reinforcement contingencies (later termed the interview-informed synthesized contingency analysis [IISCA], Jessel et al., 2016). All putative reinforcers are presented together in a single test condition and compared to a matched control condition in a multielement design. To maintain a safe and controlled environment, the reinforcers are combined into a synthesized contingency to abolish all motivation to engage in any challenging behavior and presented contingent on any associated nondangerous behavior (e.g., whining) to reduce the potential for escalation to dangerous behavior (e.g., aggression). The total time to conduct the IISCA is around 30 min (Jessel et al., 2016).

Skill-based treatment (SBT; Hanley et al., 2014) typically follows the PFA as part of a comprehensive assessment and treatment process. SBT consists of teaching communication, toleration, and cooperation skills using the same synthesized contingency identified during the IISCA. The PFA and SBT package has robust existing support in the literature (e.g., Coffey et al., 2021; Layman et al., 2023). However, procedural modifications (e.g., latency-based IISCA, trial-based IISCA, single-session IISCA) have been made to the IISCA to address clinicians’ needs (e.g., more efficient and ecologically relevant assessment methods; see Metras & Jessel, 2021 for more on procedural modifications) as well as attempting to be more aligned with the principles of trauma-informed care (TIC; Rajaraman et al., 2022a). Specifically, the performance-based IISCA incorporates a trauma-informed framework (Jessel et al., 2024).

Iovino et al. (2022) conducted one of the first demonstrations of the performance-based IISCA with five Italian children with autism. Differentiated outcomes supporting a functional relation between challenging behavior and synthesized reinforcement were obtained for all five participants; however, the study concluded after the performance-based IISCA and there was no demonstration of treatment effects reducing challenging behavior following the assessment. Jessel et al. (2024) extended the research on the performance-based IISCA by comparing the results of 12 applications of the performance-based IISCA to a subset of 7 applications of the original IISCA before implementing function-based treatment (i.e., functional communication training [FCT]) with 5 participants who experienced both IISCA formats. Function-based treatment successfully decreased challenging behavior based on the results of the performance-based IISCAs. Thus, Jessel et al. confirmed strong correspondence with the performance-based IISCA and the original IISCA format. Furthermore, the authors demonstrated that the results of the performance-based IISCA could be used in the design of a function-based treatment to yield reductions in challenging behavior, as well as increases in communication skills. To our knowledge, Iovino et al. (2022) and Jessel et al. (2024) are the only empirical demonstrations of the performance-based IISCA. Thus, more studies are needed to evaluate the performance-based IISCA.

Recent calls to consider TIC in the design and delivery of behavioral services are relevant to functional analysis arrangements. Rajaraman et al. (2022a) proposed a framework for incorporating TIC into behavior analytic services: (a) acknowledging a client’s potential traumatic history; (b) creating an environment that promotes safety and trust through rapport building and maintenance; (c) arranging choice making opportunities to promote shared governance and client autonomy; and (d) teaching adaptive skills. The authors highlighted the importance of taking a proactive approach to reduce the possibility of retraumatizing individuals while rendering services, in part by pointing out that many individuals with autism who exhibit challenging behavior are at increased risk of experiencing potentially traumatic events. One suggestion for doing so was to align specific procedures of the assessment and intervention process with their proposed trauma-informed framework as a guide. Behavior-analytic researchers have recently begun this type of alignment in empirical studies, providing a foundation for further evaluation of procedures aligned with TIC (e.g., Gover et al., 2023, 2024; Jessel et al., 2024; Pollack et al., 2024; Rees et al., 2023).

The performance-based IISCA uniquely aligns with TIC principles in multiple ways. First, the clinician acknowledges the individual’s potential trauma by reducing exposure to dangerous behavior and the evocative events that contribute to the behavior. The entire functional analysis can be completed with the clinician observing as little as three or five instances of associated nondangerous behavior and the clinician strives to minimize deliberate exposure to adverse events. Second, the clinician attempts to ensure the child’s safety and trust by extending access to a synthesis of rich reinforcement until the individual is calm and behaviorally prepared for the removal of those preferred items and events. That is, the clinician does not ever remove reinforcers when the individual is visibly distressed and likely to escalate to exhibiting dangerous behavior. Third, the individual has the choice to withdraw assent at any time during their experience with the performance-based IISCA procedures.

Although Jessel et al. (2024) obtained efficacious outcomes with respect to FCT informed by the performance-based IISCA, whether such a starting point could inform a more comprehensive intervention remains to be explored. In addition, a key pillar of the trauma-informed framework is the focus on skill-building during treatment (Rajaraman et al., 2022a). FCT, a behavioral intervention focused solely on teaching communication skills, may not be fully representative of the greater expectations of a trauma-informed approach (i.e., one that teaches multiple skills of varying complexity and difficulty to be used in varied evocative contexts).

The purpose of this study was threefold. First, we sought to replicate the procedures and outcomes of the performance-based IISCA with three children with autism. The literature on the performance-based IISCA remains sparse and systematic replication is essential. Second, we conducted the first SBT evaluation informed by the performance-based IISCA in which we taught multiple skills including communication, toleration, and cooperation, while thinning reinforcement. It is currently unknown if reinforcement can be successfully thinned and a comprehensive set of skills can be taught using the results of the performance-based IISCA. In addition, two of the three participants experienced sessions with their caregivers as implementers after reinforcement was thinned, supporting some generality of the treatment. A third purpose of our study was to acquire information about the social validity of the procedures and outcomes of the entire PFA/SBT process, as this has not been included in previous studies evaluating the performance-based IISCA. Beyond proving that a treatment is efficacious in its goal to reduce challenging behavior, it is important to ensure that it is also perceived by constituents of the services to be safe, helpful, and acceptable.

## Method

### Participants and Settings

Three participants were referred to an outpatient clinic that specialized in the assessment and treatment of dangerous behavior. The caregivers scheduled two to three 1-hr visits per week and all services (from intake to discharge) were completed within approximately three months for all participants. The session rooms were approximately 3 m by 3 m. Each room had a padded play area where the child had access to preferred items. The rooms also had a table and two chairs to complete tasks or academic work. Each room was equipped with a camera that was live-streamed during each phase of the PFA and SBT, and caregivers could view the livestream from the waiting room. We attempted to include the family in the decision-making process by encouraging them to ask questions and provide feedback while watching every session live throughout the process. The door of the therapy room was always left open during each phase of the PFA and SBT, allowing the child to leave and go to the clinic waiting room with their caregivers at any time. This did not occur during any of the performance-based IISCAs.

Rich^1^ was a 9-year-old, European American boy diagnosed with ASD, attention-deficit/hyperactivity disorder, a language and speech disorder, and a learning disability. His vocal abilities included 1-word utterances. Rich was homeschooled due to the severity of his challenging behavior. Rich’s mother reported that when the doors of their home were closed, Rich would become visibly distressed and punch a hole through the doors. Doors remained open at all times in their home to decrease the chances of evoking dangerous behavior. In their everyday life, Rich’s mother had to remain by Rich’s side at all times because he would engage in challenging behavior if they separated. He engaged in dangerous behavior including aggression, disruptive behavior, and SIB, and associated nondangerous behavior including physical intimidation. His aggression involved using closed or open fists to hit others, SIB involved hitting his head with a closed fist, and property destruction involved tearing and throwing items. His physical intimidation involved raising a closed fist above his head and taking a step toward another person.

Troy was a 5-year-old, African American boy diagnosed with ASD. He communicated using brief, disfluent sentences. Troy’s mother reported that Troy would often exhibit extended tantrums that could last for hours. During these tantrums, Troy would elope to break or tear any items he could reach. If others were around him during an extended tantrum, he would aggress toward them; however, Troy would engage in SIB, by using his closed fist to hit his head, if no one was within arm’s reach. Troy’s mother reported that it was often difficult to manage his dangerous behavior and she was afraid for his safety as well as the safety of others around him, including his other young siblings.

Chung was a 4-year-old, Chinese American boy diagnosed with ASD. Although Chung could make some verbal approximations when vocally prompted, he did not exhibit any independent vocal communication skills. Caregivers reported his dangerous behavior as aggression, which involved scratching faces and pulling the hair of others. Caregivers reported his most difficult behavior to manage involved extended tantrums where he would begin to swipe or throw items and engage in SIB such as scratching himself while rolling on the floor screaming and crying. These extended tantrums would often cause him to have nosebleeds.

### Measurement

During the participants’ first visit to the clinic, the caregivers were asked to complete questionnaires and a consent form to participate in research. The questionnaires included the Aberrant Behavior Checklist Second Edition (ABC-2; Aman & Singh, 2017) and the Parenting Stress Index Fourth Edition Short Form (PSI-4-SF; Abidin, 2012). These were administered to collect measurements and to include family members in the therapeutic process. The ABC-2 is a questionnaire that delineates between four subscales of challenging behavior. The clinician converted the raw scores from the completed ABC-2 forms into percentile rankings for youth with ASD (see p. 84 of ABC-2 manual). The PSI-4-SF categorizes parental stress into three domains: parental stress, parent–child dysfunctional interaction, and difficult child. The scores from each of the domains are summed to create a total stress value and create a percentile profile. Both questionnaires, the ABC-2 and the PSI-4-SF, were completed by caregivers during intake and again before discharge.

To ensure safety, we measured and targeted dangerous and nondangerous topographies of challenging behavior in an open-contingency class during the assessment and treatment process. Dangerous and associated nondangerous behavior were only included in the open-contingency class if they were reported by caregivers to be functionally similar (Warner et al., 2020). We considered dangerous behavior to be any topography that posed a serious risk or resulted in injury to oneself or others (e.g., SIB, aggression, property destruction). We considered any associated nondangerous behavior as any topography that did not result in injury to oneself or others (e.g., whining, crying, inappropriate vocalizations, physical intimidation). The performance-based IISCAs display the count of challenging behavior and distinguishes between dangerous and nondangerous behavior. All challenging behavior (dangerous or nondangerous) was aggregated into a single measure during the treatment evaluation and calculated as a rate by dividing the frequency by the session duration.

We measured two alternative behaviors, calmness and engagement, as durations during the performance-based IISCA. We also measured reinforcement as a duration, beginning with the termination of the establishing operation and the presentation of preferred items or events (i.e., synthesized reinforcement context). We converted the duration measures to a percentage of the session by dividing the duration of the behavior by the session time. Other alternative behaviors that were measured or targeted included interactive behavior, functional communication responses (FCRs), tolerance responses, and cooperation.

The count of interactive behavior was only included in the performance-based IISCAs. FCRs were measurements of the participants’ communication skills and we distinguished between simple FCRs, intermediary FCRs, and complex FCRs. The rate of communication and tolerance responses was reported by dividing the count of the FCR or tolerance response by the session duration.

We chose the instructions and tasks that targeted cooperation based on the participant’s developmental level and individual goals, which were co-developed with caregivers during the open-ended interview and throughout SBT. We reported cooperation as a percentage by dividing the number of tasks completed by the number of instructions given and multiplying the quotient by 100. A summary of dangerous behavior, nondangerous behavior, calmness, engagement, interactive behavior, and cooperation is presented in Table 1. The FCRs and tolerance responses for each participant are presented in Table 2.

**Table 1:** Summary of dependent variables

Response class	Topographies	Description
Dangerous challenging behavior	SIB, aggression, disruptive behavior	Any topography of behavior that posed a serious risk or resulted in injury to oneself or others
Nondangerous challenging behavior	Whining, crying, inappropriate vocalizations, physical intimidation	Any topography of behavior associated with dangerous behavior that did not result in injury to oneself or others
Alternative behavior	Calmness	A neutral or positive affect without any idiosyncratic indications of distress (e.g., excessive stereotypy, rocking, challenging behavior)
	Engagement	Directing their attention to their reinforcers, preferred items, or activities (e.g., watching a video, arranging items)
	Interactive Behavior	Any initiation made to engage with the clinician (e.g., “Come watch this video with me,” asking for tickles)
	Cooperation	Following adult instructions and engaging in contextually appropriate behaviors

*Note. *SIB refers to self-injurious behavior

**Table 2:** Summary of functional communication and tolerance responses

Participant	Simple FCR	Intermediary FCR	Complex FCR	Tolerance response
Rich	“My”	–	“My way”	“Okay”
Troy	“My way”	“My way, please”	“Excuse me, my way please”	“Okay”
Chung	4 × 4 in. icon placed in front of him	2 × 2 in. icon within arms-reach but off to his side	2 × 2 in. icon on top of a communication booklet placed in a location that required him to scan his surroundings to locate	Another 2 × 2 in. icon located inside of his communication binder

*Note. *FCR refers to functional communication response

### Interobserver Agreement (IOA)

A secondary observer collected interobserver agreement (IOA) on all of the participants’ performance-based IISCAs and at least 30% of the treatment evaluations. We calculated a partial interval agreement by dividing each session into 10-s intervals and dividing the smaller number of the data collected by the larger number and multiplying that average by 100 to obtain a percentage. We calculated IOA for all challenging behavior (nondangerous and dangerous), appropriate behavior (interactive behavior, calmness, engagement, FCRs, tolerance, and cooperation), and reinforcement. The mean IOA for all participants was above 90%. A summary of IOA for all participants is presented in Table 3.

**Table 3:** Interobserver agreement obtained during the performance-based IISCA and treatment evaluation

*Performance-based IISCA*						
	Challenging behavior		Appropriate behavior			Reinforcement
	Nondangerous	Dangerous	Interactive behavior	Calmness	Engagement	
Rich	91%	100%	95%	93%	96%	96%
Troy	97%	100%	100%	91%	90%	90%
Chung	97%	100%	100%	98%	95%	95%
*Treatment Evaluation*						
	Nondangerous	Dangerous	FCRs	Tolerance	Cooperation	
Rich	98% (78–100)	100%	97% (76–100)	93% (79–100)	94% (85–100)	97% (94– 99%)
Troy	100%	100%	99% (89–100%)	100%	99% (92–100%)	96% (86–100%)
Chung	99% (93–100%)	99% (96–100%)	99% (88–100%)	98% (91–100%)	97% (88–100%)	94% (76–99%)

*Note. *Numbers in parentheses represent the range. *FCR* refers to functional communication response

### Experimental Design

The frequency of challenging behavior when the reinforcers were absent was compared to the frequency of challenging behavior when the reinforcers were present during the performance-based IISCA. Experimental control was demonstrated when instances of challenging behavior occurred more when the reinforcers were absent and the establishing operation was present. We conducted the SBT treatment validation using a multiple baseline across responses with a brief return to baseline following the first phase of FCT. Experimental control was demonstrated when challenging behavior increased during the return to baseline and was eliminated or decreased once FCT was reinstated. Functional control of FCRs was demonstrated with the elevated rates of the appropriate FCR during each phase.

### Procedures

#### Open-Ended Interview

The PFA began with an open-ended interview conducted with the caregivers (see appendix of Hanley, 2012). The open-ended interview included questions about the (a) participants’ language abilities and preferences, (b) topographies of challenging behavior, and (c) context in which challenging behavior had occurred. The questions regarding language abilities were used to categorize the participants’ baseline abilities (i.e., nonspeaking, 1-word utterances, disfluent sentences, fluent sentences) and helped the clinician determine what simple communication to begin teaching once the treatment was introduced. The clinician used the questions regarding topographies of challenging behavior to identify an open-contingency class targeting a collection of responses the caregivers had experienced ranging from dangerous (e.g., aggression, property destruction, SIB) to precursors to dangerous behavior (e.g., screaming, whining, yelling, swearing). All the topographies described by the caregivers to be functionally related were then included within the open-contingency class. The remaining questions regarding the context in which challenging behavior occurred were most important for designing the performance-based IISCA. The clinician asked caregivers questions about any potential antecedents that were likely to evoke challenging behavior and consequences that were likely to de-escalate behavior. The answers from the caregivers helped the clinician develop a unique contingency representative of the context in which the caregivers were likely to experience challenging behavior in the home, school, or community. Developing a unique contingency based on the caregiver’s answers was important not only for ecological validity but also for following the trauma-informed guideline of including family and shared governance (Pollack et al., 2024). This unique contingency, including preferred and evocative events, was then evaluated in the performance-based IISCA.

The open-ended interview identified preferred and evocative events to be assessed during the performance-based IISCA for all participants. Rich’s preferred events included independent play with his phone and tablet. Interrupting this play with any sort of requests or instructions was identified as the evocative event. Troy preferred to play independently with a tablet and boxes of various alphabet letters. The evocative event included academic work completed at the table. The preferred events for Chung included interactive play with activities such as kinetic sand, marble towers, and dinosaurs. The evocative event included discrete trial instructions at the table.

The open-ended interview required approximately 15 min to conduct with each caregiver. During this time, the participant was provided with noncontingent access to preferred events and any reasonable requests were honored to build a healthy relationship between the participant and clinic assistant. The clinician brought the caregiver to a separate room and procedures were implemented to promote the participants’ comfort and safety. For Chung, the door of the interview room was left ajar so that he could see his caregivers and choose to leave and spend time with his caregiver at any point during the interview. To ensure safety, the clinician conducted the open-ended interview with both Rich and his mother in the same room to prevent distress that might be caused by separation from his mother. Troy displayed an initial discomfort leaving his father’s side; however, after a few min of playing with the clinic assistants, the father was able to leave the room for the open-ended interview and Troy continued to play with the clinic assistants without incident.

### Performance-based, Interview-Informed Synthesized Contingency Analysis (IISCA)

Prior to the performance-based IISCA, the clinician provided three min of noncontingent access to the preferred activities identified during the open-ended interview to build rapport and ensure the participant was comfortable before beginning the analysis. If challenging behavior consistently occurred during this time, the clinician would have discontinued the session and returned to open-ended questioning with the caregiver. This did not occur with any of the participants. The clinician initiated the performance-based IISCA as long as the participant was calm during the final 30 s of access to the preferred activities.

The performance-based IISCA began with the simultaneous removal of the preferred activities and the presentation of the individualized evocative events. This change in the environment often coincided with an instruction, such as, “Playtime is all finished, it is time to do some work.” The evocative events continued to progress if the participant cooperated with all instructions (i.e., instruction to stand up, then walk to the table, then begin completing work) until 10 min had elapsed. If no challenging behavior occurred within the 10 min, the performance-based IISCA would have been discontinued and the clinician would have asked the caregiver additional open-ended questions about other potential evocative events. This did not occur. The clinician reinstated access to all reinforcers immediately following the first instance of any nondangerous or dangerous challenging behavior. The clinician also provided a statement of support (e.g., “It is okay, we can play. Don’t worry.”). The preferred events were then available for 30 s after the onset of calm behavior. After 30 s of calm behavior, the evocative events were re-presented. Any challenging behavior or any indices the participant was not calm during access to the preferred events resulted in the 30-s timer being reset.

The performance-based IISCA was discontinued after 5–6 instances of challenging behavior corresponding to 5–6 evocative events. The criteria for ending the performance-based IISCA was the presentation of five consecutive establishing operations that successfully evoked challenging behavior and the de-escalation of behavior following the reinstatement of synthesized reinforcement. There was no set minimum duration and the clinician interpreted the results in real-time with a differentiated outcome indicated by challenging behavior reliably occurring during periods in which the evocative events were present and not occurring when the participant had access to the preferred events.

### Baseline

The clinician began SBT once the performance-based IISCA empirically identified caregiver-informed evocative events that reliably evoked challenging behavior and preferred events that reliably de-escalated behavior. The baseline phase was similar to that of the performance-based IISCA in that the evocative events were presented and any challenging behavior resulted in the simultaneous removal of the evocative events and presentation of the preferred activities. Baseline sessions were 3 min.

### Functional Communication Training (FCT)

The first phase of SBT included FCT to teach increasingly complex responses (Ghaemmaghami et al., 2018). The complexity of responses increased from the simple FCR to the terminal FCR by adding to the response effort of the FCR through additional letters or words, social niceties (e.g., “Please”), and social interactions. The variables added to the complexity of responses were determined by the participant’s communication abilities and progression during FCT. The clinician selected a simple FCR that was at or below the baseline abilities identified during the open-ended interview. The intermediary FCR extended the sentence structure or added additional nonverbal response requirements (for those who were nonspeaking). The terminal complex FCR included conversational niceties along with an interaction with the clinician. For example, Troy’s complex FCR involved him first saying “Excuse me” in order to get the attention of the clinician. Once the clinician acknowledged Troy, his complex FCR continued with, “My way, please” before the clinician returned access to the reinforcers for 30 s.

The clinician taught the target communication response during training prior to each treatment evaluation phase. Teaching Chung to emit his FCRs (i.e., 4 by 4 in. and 2 by 2 in. communication cards) involved the clinician using a most-to-least prompting hierarchy to show Chung how to place the communication card in the clinician’s hand. The clinician began with an immediate physical prompt to use the communication card and began fading the prompt to partial physical and gestural prompts. The clinician used a full verbal prompt for Rich and Troy and gradually faded the verbal prompt (e.g., *My way, my w…, my…, m…*) while inserting a time delay of 2 to 5 s. The clinician provided 30-s access to individualized reinforcers for prompted or independent communication. If the participant engaged in nondangerous challenging behavior during training, the establishing operation was slowly progressed, meaning we continued to introduce transitions or instructions, and a prompt was provided to emit the FCR when the participant was calm and not engaging in nondangerous challenging behavior. In the event of dangerous problem behavior during training, the participant was immediately prompted to emit their FCR. If the participant emitted the FCR following the prompt, the reinforcers were provided, regardless of the presence or absence of dangerous challenging behavior. If the FCR was not emitted following the prompt and dangerous challenging behavior continued, all reinforcers were provided immediately, to ensure safety.

Participants met criteria to begin the treatment evaluation following two training sessions of five trials with 100% independent responding and little to no challenging behavior. The only difference between training and the treatment evaluation was that the clinician no longer provided prompts. If the participant did not emit the target FCR, the clinician continued to progress the evocative events, meaning continued to instruct them to transition or work on the present task. If the participant engaged in nondangerous challenging behavior, the evocative events continued to be progressed. If they emitted a dangerous response, reinforcement was provided to ensure safety. Sessions during the FCT treatment evaluation were 3 min and the reinforcer duration was 30 s.

### Baseline Probe

Following the first phase of the FCT treatment evaluation for each participant, a baseline reversal was implemented. During the reversal, reinforcement was not provided contingent on the simple FCR. Instead, the FCR was denied and the evocative events were progressed until the participant engaged in challenging behavior. Reinforcement was provided for 30 s contingent on challenging behavior and the session was 3 min. The purpose of the reversal was to demonstrate continued functional control. It also provided additional evidence for the necessary subsequent skills to be taught in the next phase of SBT.

### Delay/Denial Tolerance Training (DDTT)

The clinician introduced delay/denial tolerance training (DDTT) following the participant successfully acquiring the complex FCR with little to no challenging behavior during the FCT treatment evaluation. If the participant was engaging in no challenging behavior or significantly less challenging behavior relative to baseline, DDTT was introduced. The clinician began DDTT by teaching the participant a tolerance response to emit following a denial to the complex FCR (e.g., “I’m sorry, we can’t right now, we need to do a little work first”). During tolerance training, the clinician reinforced the complex FCR for approximately 50% of the trials. During the other half of the trials, the clinician initiated the denial and only re-presented the reinforcers following the tolerance response. The clinician did not signal the reinforcement contingency; there were no discriminative stimuli signifying if the complex FCR was going to be reinforced or denied. Tolerance training used the same prompting strategies as FCT and included a separate treatment evaluation phase. Sessions during the tolerance treatment evaluation were 3 min and the reinforcement interval continued to be 30 s.

All skill-based teaching occurred within the same treatment evaluation contexts after the participant acquired the tolerance response and sessions were no longer time-based. In addition, the reinforcement interval was increased from 30 s to 1 min. Each session consisted of five (Rich and Troy) or six trials (Chung). Sessions with five trials consisted of (a) one trial in which the complex FCR was reinforced, (b) one trial in which the tolerance response was reinforced, and (c) three trials in which cooperation was reinforced (the six trial session included an additional trial in which the complex FCR was reinforced). Each of the three trials in which the clinician reinforced cooperation varied in the number of instructions that were provided. The cooperation trials with the least, middle, and most amount of work were designated as *easy, moderate,* and *difficult*, respectively. That is, the clinician presented reinforcement in a probabilistic fashion and no discriminative stimuli were included that signaled the response requirement to the participant. The order of the trials was determined randomly. The clinician created laminated cards, mixed them up, and selected each card until all trials for the session were determined. The clinician repeated this process for each session.

To maintain rapport, the clinician provided an empathetic statement and continued to present the evocative event if the participant exhibited an associated nondangerous behavior (e.g., “I know it’s hard but we can do this. Only a little more left!”). To avoid escalation and promote emotional safety (Rajaraman et al., 2022a), dangerous behavior resulted in the immediate discontinuation of the evocative events and a return to the preferred items. By returning reinforcers following dangerous behavior, the clinician treated these dangerous responses as a form of removing assent to participate (Breaux & Smith, 2023). Had the participant left the room entirely for more than 1 min, their choice would have been honored and the session would have been discontinued until the participant chose to return to the room on their own in a calm manner. The clinician never blocked a child from leaving the room or physically prompted the child to return to the room. Chung’s sessions were discontinued twice in this manner. This did not occur for any other participant.

Reinforcement was thinned during DDTT using a contingency-based progressive delay (Ghaemmaghami et al., 2016). The three trials incorporating instructions were gradually increased in a systematic fashion while maintaining the same distinction between easy, moderate, and difficult. For example, the clinician initially presented the participant with only one instruction to cooperate with before reinforcers were returned. Once the participant met the criteria for successfully mastering cooperation with one instruction, sometimes the participant was then presented with one instruction (easy), two instructions (moderate), or three instructions (difficult) to complete. The next reinforcement thinning step could have included two instructions (easy), three instructions (moderate), or six instructions (difficult). The thinning steps were client specific and dependent on observed comfort level with evocative events (i.e., an increase in challenging behavior resulted in the need for smaller incremental steps). The criteria for moving on to each step primarily depended on three potential factors. This included challenging behavior remaining low, appropriate and stable use of the target communication and tolerance responses, and high levels of cooperation. After Troy took a vacation and a long time had passed after the last session, the clinician repeated a step and did not introduce a new step. This only occurred for Troy (see thinning level 4 in Fig. 3).

### Novel Implementers

To include family in the therapeutic process, sessions with novel implementers were conducted after DDTT with Rich and Troy. Rich’s mother was included in every session and she agreed to conduct sessions once he had mastered all thinning steps. Troy had a novel student observer who was a volunteer for the university-based clinic and had never worked with autistic individuals before but was interested in applied behavior analysis (ABA). Troy’s father also agreed to conduct one session after training. Chung’s father agreed to be trained but did not consent to formal sessions with video recordings or data collection. All novel implementers were trained using modeling, rehearsal, and feedback to conduct the final thinning level reached with the participant. No data were collected during the training. The clinician remained in the room during sessions to provide in situ feedback and instruct the novel implementer on what trial to conduct.

### Social Validation

The clinician provided caregivers with a social validity questionnaire following the participants’ discharge from services. The questionnaire included two sections with seven questions in total. The first section included three questions regarding the acceptability, safety, and representativeness of the functional assessment procedures. The second section included four questions regarding the satisfaction with improvement in challenging behavior and communication, helpfulness, and acceptability of the treatment procedures. Caregivers answered each question on a Likert scale ranging from 1 (lowest score) to 7 (highest score). Each question also included room for any open-ended comments and space was provided at the end of the questionnaire for any additional comments.

## Results

### Practical Functional Assessment (PFA)

**Fig. 1:**
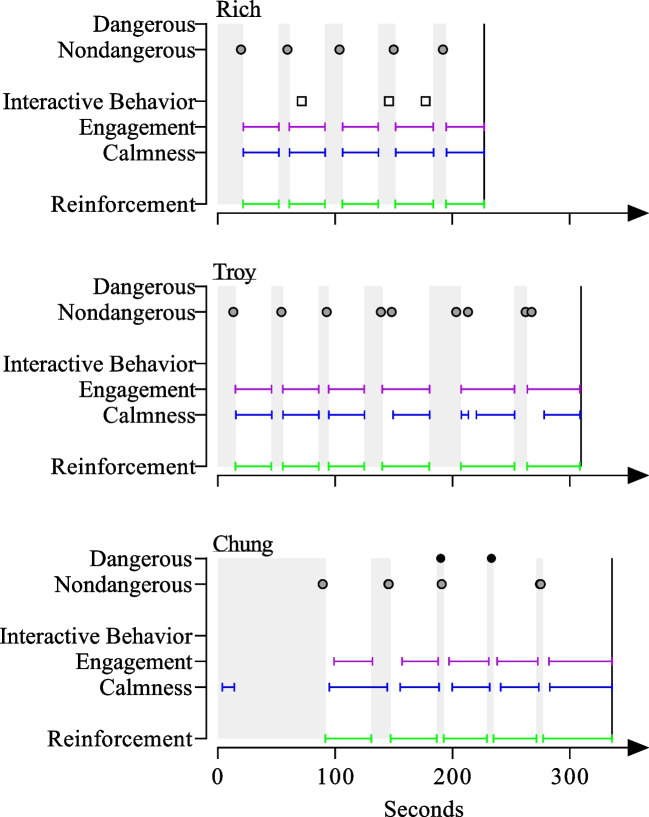
Results of the performance-based IISCA. *Note*. Shaded area represents the reinforcer absent period. The vertical line represents the end of the performance-based IISCA

Figure 1 displays the results of the performance-based IISCAs. Rich (top panel) only exhibited challenging behavior when evocative events were present and all instances were associated nondangerous behavior. Rich immediately resumed calm and engaged behavior during access to the preferred items. Functional control was deemed sufficiently demonstrated after five instances of challenging behavior occurring during five evocative event presentations and the performance-based IISCA was discontinued after 227 s (3.78 min).

Troy (middle panel) also did not exhibit any dangerous behavior during the performance-based IISCA. Associated nondangerous behavior began occurring during the presentation of evocative events. However, Troy continued to engage in one instance of challenging behavior (i.e., whining) after the evocative events were terminated and reinforcers were returned. In other words, despite the immediate presentation of reinforcers contingent on whining, it took some time for him to return to a calm state without challenging behavior. The clinician conducted one additional evocative event, totaling six presentations of the evocative events during Troy’s performance-based IISCA, to determine if this pattern would continue. The clinician discontinued the performance-based IISCA after 308 s (5.13 min) and determined that Troy’s problem behavior did not immediately “turn off” with the presentation of synthesized reinforcement. Despite this delay to a return of calmness, the results still supported a demonstration of functional control because Troy engaged in more challenging behavior during the presentation of evocative events relative to the presentation of reinforcers.

All of Chung’s (bottom panel) challenging behavior occurred during the presentation of evocative events. Most instances were of associated nondangerous behaviors with some exceptions. Initially, Chung did display some calm behavior during the first two presentations of the evocative events. By the third evocative event, Chung immediately exhibited challenging behavior and was more reliably calm during access to the preferred events. The clinician determined functional control to be demonstrated after five evocative event presentations and discontinued the performance-based IISCA after 336 s (5.6 min).

### Skill-based Treatment (SBT)

**Fig. 2:**
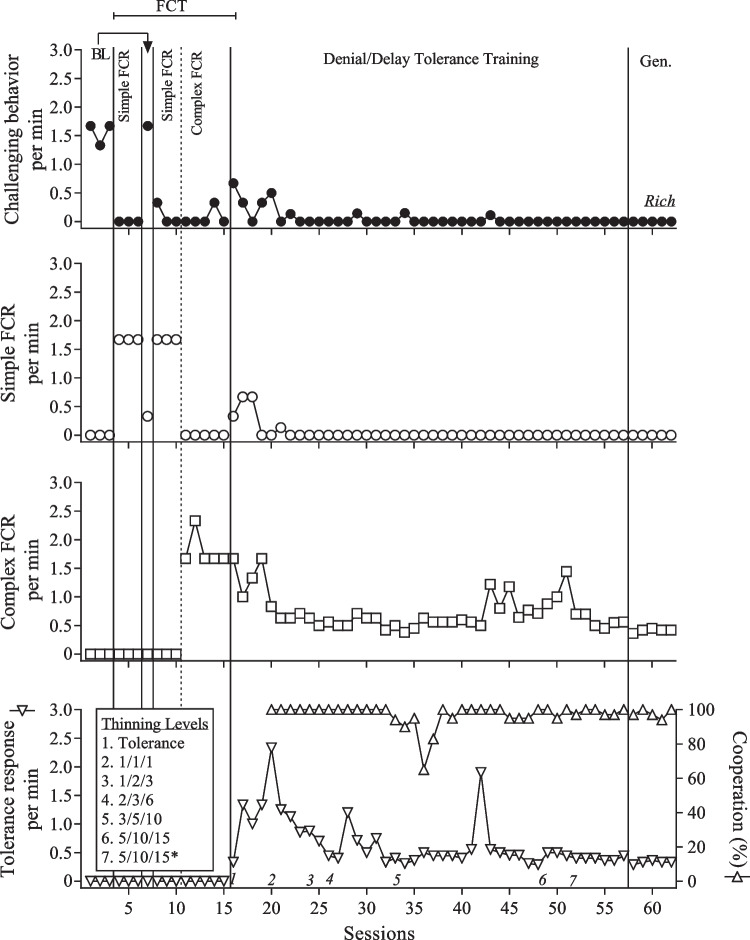
Results of the treatment evaluation for Rich. *Note*. The asterisk indicates that the thinning level includes transitions. Gen. refers to generality

Figure 2 represents the treatment evaluation for Rich. Challenging behavior occurred during the initial baseline phase (*M* = 1.56 responses per min [RPM]; *SD* 0.2). Once reinforcement was arranged for the simple FCR, challenging behavior was eliminated and the simple FCR began to occur (*M* = 1.67 RPM; *SD* 0). The return to baseline resulted in an increase in challenging behavior and a decrease in the simple FCR. The clinician continued FCT returning to the simple FCR (*M* = 1.67 RPM; *SD* 0) and challenging behavior decreased (*M* = 0.11 RPM; *SD* 0.19). A complex FCR (*M* = 1.8 RPM; *SD* 0.3) was taught in the final phase of FCT and challenging behavior remained low (*M* = 0.07 RPM; *SD* 0.15). Once the clinician began teaching the tolerance response, there was a slight re-emergence of challenging behavior and the simple FCR; however, the complex FCR continued to occur at elevated rates relative to baseline. Challenging behavior remained low (*M* = 0.06; *SD* 0.14) as the clinician thinned reinforcement during DDTT and did not occur at all during sessions with his mother. Cooperation throughout DDTT remained high (*M* = 97.23%; *SD* 6.08).

**Fig. 3:**
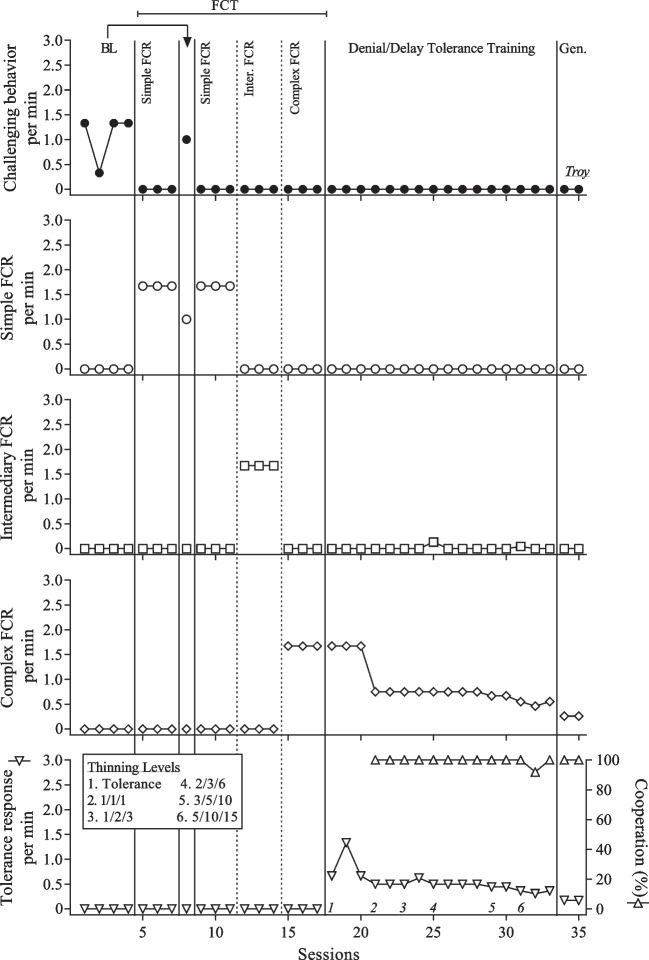
Results of the treatment evaluation for Troy. *Note*. Gen. refers to generality

The results of Troy’s treatment evaluation are presented in Fig. 3. Challenging behavior was elevated during baseline (*M* = 1.08 RPM; *SD* 0.5) and no communication occurred. During the teaching of the simple FCR, challenging behavior was eliminated and the simple FCR began to occur at elevated rates (*M* = 1.67 RPM; *SD* 0). The return to baseline resulted in the re-emergence of challenging behavior and a slight decrease in the simple FCR. Challenging behavior remained eliminated throughout the remainder of the treatment evaluation, including during sessions with the student implementer (one session) and the father (one session). The target communication reliably occurred when it was reinforced and cooperation was high throughout DDTT (*M* = 99.47%; *SD* 2.07).

**Fig. 4:**
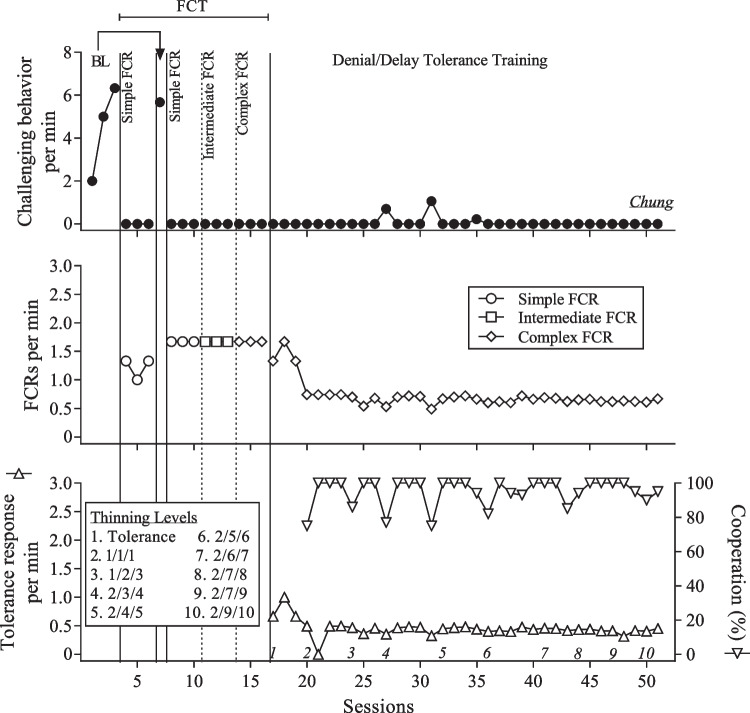
Results of the treatment evaluation for Chung

Figure 4 displays the results of Chung’s treatment evaluation. Challenging behavior occurred at an increasing rate during the initial baseline (*M* = 4.44 RPM; *SD* 2.22). Simple FCRs began to occur at elevated levels compared to baseline (*M* = 1.22 RPM; *SD* 0.19) and challenging behavior was eliminated throughout the entire FCT phase. The intermediary and complex FCRs occurred at identical rates (*M* = 1.67 RPM; *SD* 0) when one replaced the other during FCT (Chung was nonspeaking and only had one communication response available at a time). Challenging behavior remained low (*M* = 0.06 RPM; *SD* 0.21) and cooperation remained high (*M* = 94.84%; *SD* 7.99) throughout DDTT. The complex FCRs (*M* = 0.73 RPM; *SD* 0.24) and tolerance responses (*M* = 0.45 RPM; *SD* 0.14) continued to occur at low and stable levels.

### Aberrant Behavior Checklist Second Edition (ABC-2)

**Fig. 5:**
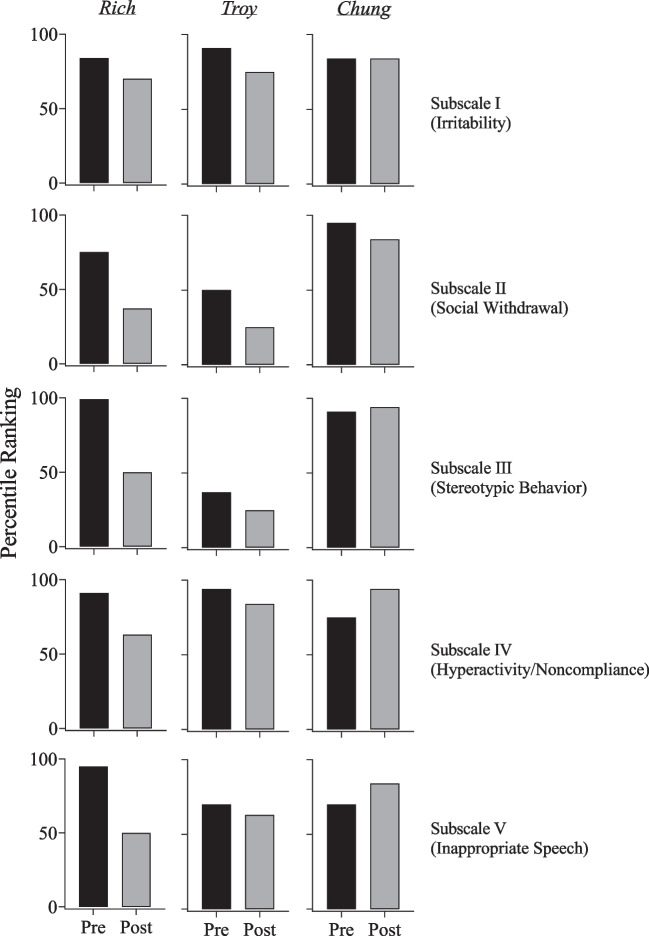
Aberrant Behavior Checklist Second Edition percentile rankings pre- and post-assessment and treatment

The results of the ABC-2 for each participant are presented in Fig. 5. The ABC-2 was completed by each participant’s caregiver prior to the PFA and again after SBT was completed. Prior to the assessment, the ABC-2 ranked Rich’s irritability in the 84th percentile. His irritability ranking decreased to the 70th percentile after treatment. Rich’s social withdrawal ranking decreased from the 75th percentile to the 37th percentile. His stereotypic behavior ranking decreased from the 99th percentile to the 50th percentile. His hyperactivity/noncompliance ranking decreased from the 91st percentile to the 63rd percentile. Lastly, Rich’s inappropriate speech ranking decreased from the 95th percentile to the 50th percentile upon completion of the PFA and SBT.

Prior to the PFA, the ABC-2 ranked Troy’s irritability in the 91st percentile. His irritability ranking decreased to the 75th percentile after treatment. His social withdrawal ranking decreased from the 50th percentile to the 25th percentile. Troy’s stereotypic behavior ranking decreased from the 37th percentile to the 25th percentile. His hyperactivity/noncompliance ranking decreased from the 94th percentile to the 84th percentile. Lastly, Troy’s inappropriate speech ranking decreased from the 70th percentile to the 63rd percentile.

Prior to the PFA, the ABC-2 ranked Chung’s irritability in the 84th percentile. His irritability was ranked the same after treatment. His social withdrawal ranking decreased from the 95th percentile to the 84th percentile. Chung’s stereotypic behavior ranking slightly increased from the 91st percentile to the 94th percentile. His hyperactivity/noncompliance ranking increased from the 75th percentile to the 94th percentile. Lastly, Chung’s inappropriate speech ranking increased from the 70th percentile to the 84th percentile.

### Parenting Stress Index Fourth Edition Short Form (PSI-4-SF)

**Fig. 6:**
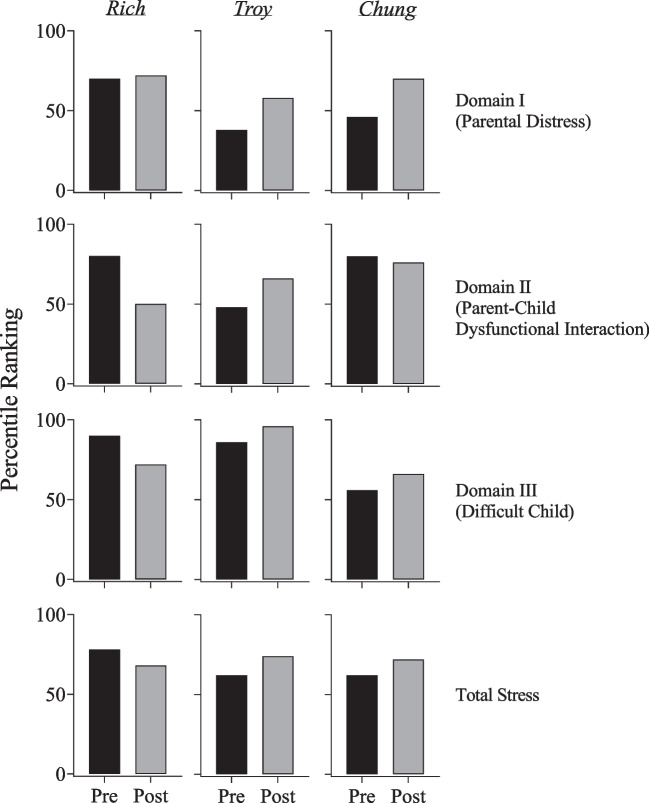
Parenting Stress Index Fourth Edition Short Form percentile rankings pre- and post-assessment and treatment

The results of the PSI-4-SF for each participant are presented in Fig. 6. The PSI-4-SF was completed by each participant’s caregiver prior to the PFA and again after SBT was completed. Prior to the PFA, the PSI-4-SF ranked Rich’s caregiver’s parental distress in the 70th percentile. Parental distress increased slightly to the 72nd percentile after SBT. Parent–child dysfunctional interaction decreased from the 80th percentile to the 50th percentile. The difficult child domain decreased from the 90th percentile to the 72nd percentile. Rich’s caregiver’s total stress decreased from the 78th percentile to the 68th percentile.

Prior to the PFA, the PSI-4-SF ranked Troy’s caregiver’s parental distress in the 38th percentile. Parental distress increased to the 58th percentile after SBT. Parent–child dysfunctional interaction increased from the 48th percentile to the 66th percentile. The difficult child domain increased from the 86th percentile to the 96th percentile. Troy’s caregiver’s total stress increased from the 62nd percentile to the 74th percentile.

Prior to the PFA, the PSI-4-SF ranked Chung’s caregiver’s parental distress in the 46th percentile. Parental distress increased to the 70th percentile after SBT. Parent–child dysfunctional interaction decreased from the 80th percentile to the 76th percentile. The difficult child domain increased from the 56th percentile to the 66th percentile. Chung’s caregiver’s total stress increased from the 62nd percentile to the 72nd percentile.

## Social Validity

The social validity questionnaire items and caregiver ratings are presented in Table 4. On the basis of the social validity questionnaire, all caregivers found the assessment to be acceptable (*M* = 6; *range* 5–7), safe (*M* = 6.33; *range* 5–7), and representative of their experiences (*M* = 5.33; *range* 4–6). In regard to the treatment, the caregivers rated that they were satisfied with the improvement in challenging behavior (*M* = 6.33; *range* 6–7) and communication skills (*M* = 6; *range* 6), they found it helpful to their home situation (*M* = 6.33; *range* 5–7), and found the procedures to be acceptable (*M* = 6.67; *range* 6–7).

**Table 4:** Social Validity Questionnaire items and results

Item	Participant			Mean
	Rich	Troy	Chung	
*Rate the extent to which you found the assessment acceptable*	7	6	5	6
*Rate the extent to which you found the assessment to be safe for your child*	7	7	5	6.33
*How well did the assessment represent the context in which you experienced problem behavior at home?*	6	6	4	5.33
*Rate the extent to which you are satisfied with the amount of improvement seen in your child’s problem behavior in our clinic*	7	6	6	6.33
*Rate the extent to which you are satisfied with the amount of improvement seen in your child’s communication skills in our clinic*	6	6	6	6
*Rate the extent to which you have found the assessment and treatment provided by our team helpful to your home situation up to this point*	7	7	5	6.33
*Rate the extent to which you have found the recommended treatment acceptable*	7	7	6	6.67

*Note*. The Likert scale for each question ranged from 1 (not acceptable, not safe, not representative, not satisfied, not helpful) to 7 (highly acceptable, very safe, very representative, highly satisfied, very helpful)

## Discussion

We were able to use the performance-based IISCA to identify synthesized contingencies maintaining challenging behavior for all three participants in less than 10 min while avoiding escalation of dangerous behavior, adding to the limited performance-based IISCA literature. Moreover, the results of the performance-based IISCA were used to inform effective treatment that taught multiple skills with little to no challenging behavior observed throughout the process. In addition to this being the first SBT evaluation of the performance-based IISCA, we included novel implementers for two of our participants. Both the procedures and outcomes received consistently high social validity ratings from caregivers, who observed all sessions and had the opportunity to implement treatment procedures. To our knowledge, this is the first socially validated demonstration of the PFA/SBT process and outcomes (Hanley et al., 2014) with the performance-based IISCA as the clinical starting point.

Given current calls to consider TIC in the design and delivery of behavior analytic research and services (Rajaraman et al., 2022a), we were mindful of how our procedures could be aligned with those principles. Although a primary goal of TIC is to minimize events that may be experienced as stressful or traumatic early in the therapeutic process, we acknowledge that we conducted additional baseline sessions prior to intervention evaluations, which prolonged exposure to potentially aversive conditions. Additional baseline sessions were conducted primarily to conform to contemporary guidelines for single-case experimental research (Kratochwill et al., 2013; Ledford et al., 2016; What Works Clearinghouse, 2020), which commonly suggest a minimum of three data points within each phase of evaluation. Furthermore, we conducted a single-session reversal to baseline conditions in each evaluation to demonstrate additional control over dependent variables by the independent variables (Ghaemmaghami et al., 2018). Although the performance-based IISCA ostensibly includes multiple replications of the effect of the synthesized contingency on various responses within a “single session,” so to speak (see Fahmie & Hanley, 2008 for a review of within-session data analysis), within-session data do not readily permit proper visual inspection when graphically depicted in the context of broader intervention evaluations for which data were aggregated to across-session measures. In other words, these additional exposures to potentially adverse conditions (i.e., baseline sessions) were included in the current evaluation to conform to research conventions that may not necessarily be germane to practice.

To the extent that increased exposure to potentially adverse conditions represents a practical or safety concern when assessing dangerous behavior, we recommend that practitioners consider progressing to intervention directly from the differentiated performance-based IISCA, and consider skipping the reversal to baseline. We believe there are sufficient opportunities to demonstrate functional control within the performance-based IISCA as well as the multiple baseline across responses design (in SBT), as there have been multiple published SBT validations that have not included reversals as a control tactic (e.g., Jessel et al., 2018; Rajaraman et al., 2022b; Rose & Beaulieu, 2019; Santiago et al., 2016). Future research could ascertain whether additional baseline sessions are needed to provide a believable demonstration of the effect of the independent variable, and could explore novel ways of graphically integrating within- and across-session data. Scheithauer et al. (2020), for example, demonstrated that additional baseline sessions did not meaningfully influence clinical decision-making or interpretations of control, and argued that functional analysis data may serve as more efficient, still sensitive baselines from which to evaluate intervention effects.

Whereas Rajaraman et al. (2022a) provided an initial framework for incorporating TIC principles into ABA research and practice, more recent publications have begun to explore how specific procedures within functional assessment and function-based interventions could be explicitly aligned with primary TIC guidelines (e.g., Gover et al., 2024; Jessel et al., 2024; Pollack et al., 2024; Rees et al., 2023). We consulted these studies to guide TIC incorporation into the current study’s procedures. For example, Pollack et al. (2024) expanded upon the framework proposed in Rajaraman et al. (2022a) and iteratively developed a trauma-informed, function-based intervention (TI-FBI) framework based upon a review of the literature on function-based interventions for students with emotional and behavioral disorders. The review conducted by Pollack et al. resulted in a large, but not exhaustive, list of practices that (a) show alignment with TIC guidelines (i.e., pillars) and (b) may be included in function-based interventions. According to Pollack et al., the aim was not to create a checklist wherein all practices would need to be incorporated to be considered trauma-informed; rather, they suggested multiple ways in which interventions could be developed in line with trauma-informed guidelines. Table 5 provides examples of some of the trauma-informed guidelines incorporated into the PFA and SBT, including *empowering children through voice and choice*, *ensuring safety,* and *teaching skills*. It is worth noting that although we reported procedural examples within the current evaluation that we argue speak to five of the six guidelines outlined by Pollack et al., we did not meaningfully *prioritize interprofessional collaboration* in the current study. Nevertheless, procedures inspired by some TIC guidelines seemed sufficient enough to produce a socially meaningful outcome across three cases. It is also important to note that many of the procedures we identified as aligned with TIC principles are common across many functional analysis formats and function-based treatments and are not specific to the PFA and SBT.

**Table 5:** Alignment with trauma**-**informed, function-based intervention framework (TI-FBI) adapted from Pollack et al. (2024)

TIC guideline	Trauma-informed practice	Present study example
Empower children through voice and choice	Obtaining the child’s assent to participate in intervention as part of the research study	An open-door policy was in place throughout the PFA and SBT, allowing the participant to exit the treatment room to indicate assent withdrawal
	Obtaining the child’s assent to participate in intervention as part of the research study	If the participant left the treatment room for more than 1 min during DDTT, the session was discontinued until they returned in a calm manner
Ensure safety	Reinforcing behaviors that precede dangerous challenging behavior	An open-contingency class of nondangerous and dangerous topographies of challenging behavior was measured and targeted during the PFA and SBT
	Synthesizing reinforcers to safely turn targeted externalizing behavior off	During the performance-based IISCA, challenging behavior resulted in the presentation of synthesized reinforcement and termination of evocative events
	Continuously monitoring participant stress responses	During the performance-based IISCA, reinforcement was not removed and the evocative event was not presented unless the participant appeared calm for 30 s
	Synthesizing reinforcers to safely turn targeted externalizing behavior off	Synthesized reinforcers were provided following the occurrence of dangerous behavior during SBT
	Providing the continuous option to leave or take a break	The participant was not physically blocked from leaving the treatment room and was not physically forced to return
Teach skills	Teaching communication skills	Participants were taught increasingly complex FCRs
	Teaching contextually appropriate behavior	Participants were taught a tolerance response
	Teaching contextually appropriate behavior	Participants were taught cooperation skills that were tailored to their family’s goals
Build healthy relationships	Fostering positive interactions between the participant and clinician	During the open-ended interview, participants were provided with noncontingent access to preferred activities and reasonable requests were honored
	Rapport-building or pairing at the start of intervention	3 min of noncontingent access to preferred events was provided to the participants prior to starting the performance-based IISCA
	Fostering connections between the participant and clinician; training the clinician to respond to participant communication attempts	During DDTT, any nondangerous challenging behavior resulted in an empathetic statement in addition to the progression of the evocative event
Include family or incorporate elements of culture and community	Seeking input from caregivers on any aspect of the intervention	The answers provided during the open-ended interview influenced the design of the performance-based IISCA
	Seeking input from caregivers on any aspect of the intervention	Caregivers viewed all sessions and were encouraged to ask questions and provide feedback during the PFA and SBT
	Seeking input from caregivers on any aspect of the intervention	Adult instructions and tasks used during SBT were co-developed in partnership with caregivers during the open-ended interview and throughout treatment
	Meaningfully involving caregivers in implementation of intervention	Two of the participants’ caregivers conducted sessions at the end of SBT to increase generality
	Seeking input from caregivers on any aspect of the intervention	The caregivers completed social validity questionnaires following discharge from services
Prioritize interprofessional collaboration	Sharing responsibility for intervention implementation among multiple support providers	–

*Note*. The language from the TI-FBI Checklist has been adapted to fit an outpatient clinic setting. Items in the left and center columns were directly adapted from Pollack et al. (2024); the right column represents novel examples from the current study. *TIC* refers to trauma-informed care; *PFA* refers to practical functional assessment; *SBT* refers to skill-based treatment; *IISCA* refers to interview-informed synthesized contingency analysis; *DDTT* refers to delay/denial tolerance training; *ABC-2* refers to Aberrant Behavior Checklist Second Edition; *PSI-4-SF* refers to Parenting Stress Index Fourth Edition Short Form. “–" indicates we did not include procedures in the current study that satisfied the guideline

Among the many TIC-associated practices reported in Table 5 and incorporated in the current study’s procedures, we believe the modified arrangement of extinction for challenging behavior warrants special consideration. Although extinction has been used in the SBT model in the past (Coffey et al., 2021; Hanley et al., 2014; Jessel et al., 2018; Rose & Beaulieu, 2019), extinction is often associated with a host of negative side effects (Lerman et al., 1999; Taylor et al., 2018) and involves the extended presentation of evocative events without reinforcement when challenging behavior is occurring. In the current study, we did not arrange for extinction of any dangerous behavior and instead programmed immediate synthesized reinforcement. This tactic was informed by the results of the performance-based IISCA, wherein any instances of dangerous behavior were immediately “turned off” when synthesized reinforcers were delivered. Although “reinforcing dangerous behavior” may appear counterintuitive to arrange in SBT, our treatment data show little to no challenging behavior across all applications, suggesting this tactic may help ensure safety while teaching important skills. This procedure shares features of what has recently been termed *kind extinction,* initially introduced by Tarbox et al. (2023) and incorporated into the procedures of Slaton et al. (2024). In avoiding the use of extinction for dangerous challenging behavior, we treated challenging behavior depending on its topography. Nondangerous instances were met with words of encouragement, potentially reducing any establishing operation related to attention as a reinforcer, but maintained establishing operations for the tangible items and escape (i.e., preferred events were not returned and instructions continued to be presented).

Incorporating the ABC-2 and PSI-4-SF allowed us to further examine the outcomes of the study from the perspective of caregivers. We used the total stress percentile ranking, which totaled the three domains of the PSI-4-SF, as the primary indicator of parental stress. Rich and Troy’s irritability ranking decreased from intake to discharge, whereas Chung’s irritability remained the same. Rich’s caregiver’s total stress decreased from intake to discharge; Troy and Chung’s caregivers’ total stress increased from intake to discharge. These outcomes might be explained by the nature of outpatient services, which might not impact homelife. Further, stress may not necessarily be amenable to immediate changes in behavior or the environment and is likely to have multiple causes, many of which would not have been addressed by the focus of this study. However, longer-term exposure to the positive effects of treatment might produce greater stress reductions. It is also possible that the timing of the second administration of the questionnaires could have influenced the results. The caregivers completed these questionnaires when treatment was completed and our services were discontinued. The process of discharge and its implications, including taking over the responsibility of intervening in their child’s challenging behavior, may have increased their stress levels. It is important to note that we cannot rule out our services as a factor in caregiver stress ratings, despite positive ratings on the social validity measure. Had we assessed social validity throughout the assessment and treatment process, we might have had more information regarding caregiver perceptions and associated stressors.

A few additional limitations also are notable and provide opportunities for future exploration. First, we did not evaluate the effects of the treatment beyond the clinic setting due to constraints imposed by our current, university-based, outpatient clinic model, wherein services are provided for free to families interested in participating in research. Although the caregivers participated in treatment implementation for two of the participants in the clinic, and although all caregivers reported that the treatment process was somewhat helpful (Chung) to very helpful (Rich and Troy) with their home situation (see Table 4), this does not imply that the same outcomes are necessarily to be expected in the home. In fact, the re-emergence of challenging behavior may be a likely occurrence when the child returns to the home context where the initial difficulties existed (Saini et al., 2018). In addition, we did not teach the novel implementers how to select and run trials, only how to implement already successful SBT procedures. Some recent publications have begun to provide a blueprint for these extensions of SBT to foster more durable outcomes and greater external validity. In one example, Rajaraman et al. (2024) described a model of in-home, telehealth-delivered PFA and SBT wherein the parent conducted all procedures and was coached to not only implement procedures but to design intervention components (i.e., how to select trials when new challenges emerge) for post-study implementation. In another example, Slaton et al. (2024) reported a year-long evaluation of SBT and demonstrated several strategies to promote and evaluate long-term, generalized improvement in the participants’ authentic learning environment. Both studies (i.e., Rajaraman et al., 2024; Slaton et al., 2024) evaluated SBT following a full IISCA, and therefore future research could incorporate some of their treatment extension strategies, with a performance-based IISCA as a starting point, to explore how to ensure that the effectiveness of a treatment is sustainable in a more natural environment.

A second limitation is that we did not collect enough treatment integrity to meet stringent research practices (i.e., specific percentage goals across phases). We recommend future research evaluate treatment integrity for the entire PFA and SBT process to ensure procedures are being implemented correctly. A third limitation is that we measured calmness and engagement only during the performance-based IISCA and did not continue measurement during treatment. Future research should address this shortcoming, as measuring engagement with reinforcers would be especially helpful during schedule thinning, and allow us to examine how engagement changes as cooperation increases. As cooperation increases, time spent in the reinforcement context will decrease, and evaluating the manner in which the participant continues to engage with their reinforcers during schedule thinning as well as their level of calmness throughout could be of value. Finally, despite our efforts to align our procedures with TIC, our participants were not screened for potential trauma histories. Future researchers may want to consider extending performance-based IISCA and SBT procedures to individuals with objective indications of trauma (e.g., diagnosis of post-traumatic stress disorder) with deference to all associated TIC principles.

## Data Availability

The data that support the findings of this study are available from the corresponding author upon reasonable request.

## Footnotes

1 Portions of Rich’s data can also be found in Jessel et al. (2024)
